# Regulatory roles of RNA binding proteins in the nervous system of *C. elegans*

**DOI:** 10.3389/fnmol.2014.00100

**Published:** 2015-01-12

**Authors:** Panid Sharifnia, Yishi Jin

**Affiliations:** ^1^Division of Biological Sciences, Neurobiology Section, University of CaliforniaSan Diego, La Jolla, CA, USA; ^2^Neurosciences Graduate Program, University of CaliforniaSan Diego, La Jolla, CA, USA; ^3^Howard Hughes Medical Institute, University of CaliforniaSan Diego, La Jolla, CA, USA

**Keywords:** RBPs, mRNA, *C. elegans*, nervous system, mRNA splicing, microRNAs

## Abstract

Neurons have evolved to employ many factors involved in the regulation of RNA processing due to their complex cellular compartments. RNA binding proteins (RBPs) are key regulators in transcription, translation, and RNA degradation. Increasing studies have shown that regulatory RNA processing is critical for the establishment, functionality, and maintenance of neural circuits. Recent advances in high-throughput transcriptomics have rapidly expanded our knowledge of the landscape of RNA regulation, but also raised the challenge for mechanistic dissection of the specific roles of RBPs in complex tissues such as the nervous system. The *C. elegans* genome encodes many RBPs conserved throughout evolution. The rich analytic tools in molecular genetics and simple neural anatomy of *C. elegans* offer advantages to define functions of genes *in vivo* at the level of a single cell. Notably, the discovery of microRNAs has had transformative effects to the understanding of neuronal development, circuit plasticity, and neurological diseases. Here we review recent studies unraveling diverse roles of RBPs in the development, function, and plasticity of *C. elegans* nervous system. We first summarize the general technologies for studying RBPs in *C. elegans*. We then focus on the roles of several RBPs that control gene- and cell-type specific production of neuronal transcripts.

## Introduction

Precise regulation of RNA, including mRNAs and small RNAs, is essential for controlling gene expression in a spatial and temporal manner. Research has identified critical roles of RBPs in neuronal development and synaptic transmission, which when disrupted through mutations, can cause neurological diseases. The *C. elegans'* genome encodes approximately 500 RBPs, defined by having an RNA binding domain such as the RNA recognition motif (RRM) and K Homology Domain (KH) (Lee and Schedl, 2006). Many of these genes are conserved from nematode to mammals, such as the PUF family of RBPs, whose name is derived from the homologs identified in *Drosophila* (Pumilio) and *C. elegans* (Fem-3) (Table 1) (MacNicol et al., 2011; Quenault et al., 2011; Dasgupta and Ladd, 2012; Colombrita et al., 2013; Modic et al., 2013; Huang and Li, 2014). Studies of RNA and RBPs in non-neuronal tissues of *C. elegans* have made pioneering discoveries for RNA interference and microRNAs (Fire et al., 1998; Lagos-Quintana et al., 2001; Lau et al., 2001; Lee and Ambros, 2001). In *C. elegans* neurons, the studies on the miRNA *lys-6* have led to a deep understanding of the complex regulatory network in neuronal fate diversification (Johnston and Hobert, 2003; Chang et al., 2004). Comparatively, the investigation for the function of RBPs in other aspects of the nervous system is just at the beginning. Recent studies by genetic screens using elegant splicing reporters in the nervous system, combined with genomic studies, have hinted at the multiple roles RBPs play in neurons, including behavior and synaptic plasticity. Here, we first review the technologies used to study RBPs in the nervous system in *C. elegans*. We then discuss several studies that have identified roles for RBPs in mRNA processing and splicing in the nervous system via mechanisms dependent on 3′-untranslated regions (3′UTRs) and small RNAs.

**Table 1:** **RBPs conserved from *C. elegans* to mammals**.

***C. elegans gene name***	**Definition**	**Target genes in neurons**	**Mammalian homolog**	**Function**
*alg-1*	Argonaute like gene-1	*lin-41*	Argonautes	Silencing, splicing, and transcriptional regulation (Huang and Li, 2014)
*ess-2*	ES2 similar-2	*dlk-1*	ES2/DGCR14	Splicing (Noma et al., 2014)
*exc-7*	Excretory canal abnormal-7	*unc-16*	ELAV-like family	Splicing, decay, translation, polyadenylatlon, transport (Colombrita et al., 2013)
*fbf-1*	Fem-3 mRNA binding factor-1	*egl-4*	PUF family	Translational regulator, localization (Quenault et al., 2011)
*grld-1*	Glutamate receptor level decreased-1	*glr-1*	Split Ends Protein (SPEN)	Splicing in *C. elegans* (Wang et al., 2010)
*mbl-1*	Muscleblind splicing regulator homolog-1	Unknown	Muscleblind	Splicing, localization, translation (Modic et al., 2013)
*mec-8*	Mechanosensory abnormality-8	*mec-2*	RNA binding protein with multiple splicing (RBPMS)	Splicing in *C. elegans* (Chalfie and Sulston, 1981; Lundquist et al., 1996; Spike et al., 2002), unclear in mammals
*msi-1*	Musashi famlly-1	*arx-1,-2,-3*	Musashi	Translational regulator (MacNicol et al., 2011)
*syd-9*	Synapse defective-9	Unknown	Zinc-Finger Protein(ZFP)	Splicing in *C. elegans* (Wang et al., 2006)
*sydn-1*	Synaptic defective enhancer-1	Unknown	Unknown	Polyadenylation regulator in *C*. *elegans* (Van Epps et al., 2010)
*unc-75*	Uncoordlnated-75	*unc-16, unc-32*, *unc-64*	CELF/BrunoL	Splicing, decay, translation (Dasgupta and Ladd, 2012)

*Listed are the gene names for RBPs in C. elegans, definition of the gene name, known targets for RBPs in the nervous system, mammaliam homologs of C. elegan RBPs, and known functions*.

## General methodology

A major technological advance in studying RNA regulation in the past decade is deep-sequencing of transcriptomes. In *C. elegans*, comprehensive transcriptome analyses for several developmental stages by the modEncode Consortium have provided valuable information for validating gene structures as well as revealing many new alternative splice junctions (Gerstein et al., 2010). An independent study that combined RNA-seq and microarray strategies also identified similar cohorts of alternative splicing transcripts as well as new isoforms of transcripts (Ramani et al., 2011). Using multiple techniques, including 3′RACE and RNA-seq, two independent studies reported global analyses of 3′UTRs in *C. elegans*, both of which revealed multiple polyadenylation and cleavage sites (PASs) in mRNA, as well as many previously unannotated 3′UTRs (Mangone et al., 2010; Jan et al., 2011). Use of multiple PAS sites may be a way to impart tissue and cell specificity of transcripts due to different isoforms.

In addition to transcriptomics, genome wide analyses of downstream targets of RBPs have been instrumental to understanding mechanisms of regulation involving RBPs, such as the miRNA induced silencing complex (miRISC). miRNAs, as part of the miRISC, guide the miRISC to specific mRNA targets. Two key components of the miRISC are the Argonautes *alg-1* and *alg-2*, which are critical for miRNA biogenesis and miRISC gene-silencing activity (Grishok et al., 2001; Zhang et al., 2007). Zisoulis et al. used the high throughput sequencing and UV cross-linking and immunoprecipitation (HITS-CLIP) method to determine the binding sites of ALG-1 (Zisoulis et al., 2010). While this study confirmed the binding sites for many previously established targets of microRNAs such as *lin-41*(Bagga et al., 2005), they also found that ALG-1 can bind along the entire mRNA transcript, with a higher percentage of occupancy toward the 3′ end of mRNA (Zisoulis et al., 2010). Interestingly, the authors found ALG-1 bound to several components of microRNA machinery, implying auto-regulation in the microRNA pathway (Zisoulis et al., 2010).

As the *C. elegans* adult hermaphrodite has 302 neurons and the major tissues are non-neuronal cells, a technical hindrance in defining transcriptomes of neurons is that neuronal mRNAs are often poorly represented in samples prepared using whole animals. Thus, a clever method, developed by Kudlow et al. allows tissue-specific CLIP in the intestine and muscle by using specific promoters tagged to the protein of interest (Kudlow et al., 2012). Than et al. adapted this approach to the nervous system by using a pan-neuronal promoter to express an epitope tagged GW182 protein AIN-2 (Than et al., 2013), one of a family of proteins that bind to the miRISC and are essential for microRNA induced silencing (Eulalio et al., 2008; Than et al., 2013). In addition to *ain-2*, *C. elegans* has another GW182 protein, AIN-1 that is also a miRISC component (Ding et al., 2005). A majority of the mRISC bound microRNA neuronal transcripts enriched in the AIN-2 CLIP assay confirmed a previous study that observed neuronal expression with these microRNAs' promoter fusion reporters (Martinez et al., 2008).

**Figure 1:**
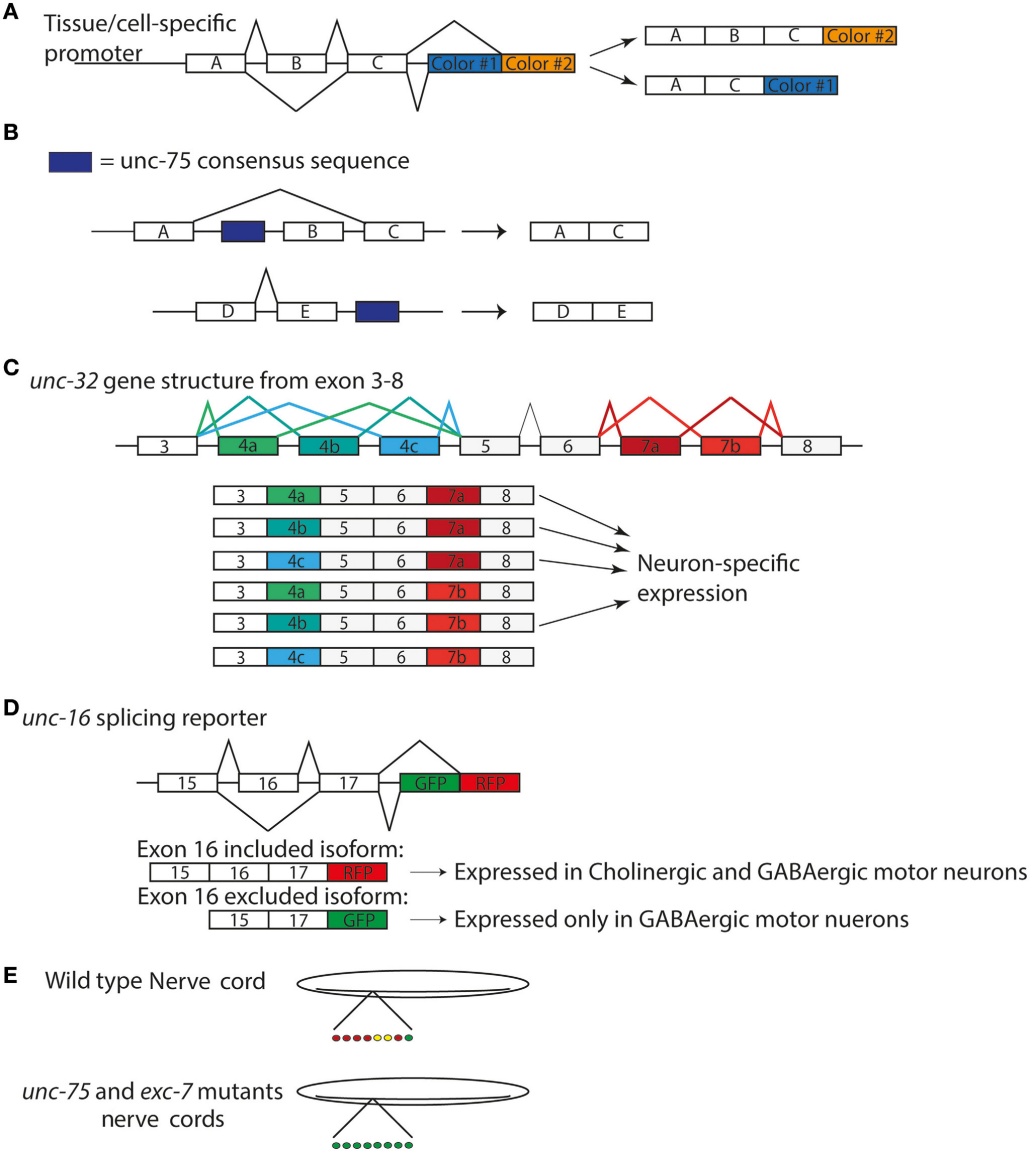
**Splicing Reporters characterize cell specific isoform expression (A)** General design of splicing reporters that change the fluorescent protein depending on the exon included/excluded **(B)** *unc-75* consensus sequence correlates with exon exclusion when upstream of exon and exon inclusion when downstream of exon (Kuroyanagi et al., 2013a) **(C)** *unc-32* gene structure displaying alternative splicing of exons 4 and 7. There are three alternative exons for exon 4 and 2 for exon 7. The six possible isoforms are shown below the gene model. **(D)** *unc-16* splicing reporter used by Norris et al. (2014) to examine expression in nervous system **(E)** patterns of *unc-16* expression motorneurons in wild type and *unc-75* and *exc-7* mutants in nerve cords (Norris et al., 2014).

Traditional genetic screening in *C. elegans* remains a powerful way to identify functionally relevant genes. Several RBPs that act in neurons were originally identified serendipitously in genetic screens for mutants disrupting specific behaviors, touch sensitivity, protein trafficking, and synapse development. Recently, the use of tissue- and cell-specific splicing reporters has also shown promise to dissect the precise regulation of neuron-type specific transcripts by RBPs at the single-cell resolution (Figure 1A). Together with the transcriptome-wide methods examining RNA regulation, genetic screens in *C. elegans* have expanded our knowledge of RBPs in the nervous system.

## Regulatory factors controlling alternative splicing of neuronal genes

Proteins involved in mRNA splicing play critical roles in regulating the maturation of transcripts. It is thought that certain neuronal diseases result from abnormal regulation of the mRNA splicing, such as in spinal muscular atrophy (SMA). One hypothesis for the mechanism of SMA is the loss of the survival motor neuron 1 protein (SMN1), which can assemble pre-mRNA splicing machinery, resulting in abnormal mRNA splicing in neurons (Burghes and Beattie, 2009).

One of the first RBPs reported to function in the nervous system of *C. elegans* was identified based on the behavioral phenotype of genetic mutants. In *C. elegans*, touch sensitivity is controlled by the mechanosensory neurons. Chalfie and Sulston carried out a forward genetic screen after random mutagenesis to find mutants displaying touch insensitivity, and identified *mec-8* (Chalfie and Sulston, 1981). Fifteen years later, Lundquist et al. cloned the *mec-8* gene and found that it contained two RRM domains (Lundquist et al., 1996). MEC-8 is broadly expressed in nervous system and epidermis. Interestingly, *mec-8(lf)* mutations genetically enhance less severe loss of function mutations of *unc-52*, which, through alternative splicing, produces multiple isoforms of perlecan, an extracellular matrix heparan sulfate proteoglycan (HSPG) conserved to humans (Lundquist et al., 1996; Spike et al., 2002). *unc-52(lf)* shows lethality at the 2-fold embryo state and appears paralyzed but certain alleles of *unc-52* are viable and fertile (Gilchrist and Moerman, 1992). In combination with *mec-8(lf)*, these viable alleles of *unc-52* mimic the lethal and paralyzed phenotype (Lundquist and Herman, 1994). Furthermore, Lundquist and colleagues found that *mec-8(lf)* results in differential expression of specific isoforms of *unc-52* by RT-PCR and immunofluorescence compared to wild type, supporting the notion MEC-8 may act as a splicing factor of *unc-52* mRNA in the epidermis (Lundquist et al., 1996). In touch neurons, Calixto et al. found *mec-8* necessary for the alternative splicing of a specific isoform of *mec-2*, a component of a degenerin/epithelial sodium channel (DEG/ENaC) involved in mechanosensation (Goodman et al., 2002; Calixto et al., 2010). These studies illustrate a single factor *mec-8* can mediate mRNA splicing of different genes in a tissue type specific manner.

The CELF/BrunoL proteins are defined by 3 RRM motifs and are conserved from *C. elegans* to humans (Dasgupta and Ladd, 2012). *In vitro* studies have shown that these proteins act mainly as splice factors (Dasgupta and Ladd, 2012). A *C. elegans* CELF protein, *unc-75*, was identified first based on locomotion behavior and later shown to affect the axonal morphology of GABAergic motor neurons (Loria et al., 2003). UNC-75 protein is localized in the nucleus, exhibiting speckles, resembling other proteins involved in mRNA splicing (Loria et al., 2003). *unc-75*'s locomotion phenotype suggests defects in neurotransmission. In *C. elegans*, presynaptic and postsynaptic phenotypes can be examined through two assays, the aldicarb and levamisole tests (Rand, 2007). The *unc-75* mutant displayed defects in the aldicarb assay but normal behavior in the presence of levamisole, suggesting a presynaptic neurotransmission role for *unc-75* (Loria et al., 2003). Importantly, this study showed that expressing vertebrate CELF4 can rescue some defects of *unc-75* mutants, supporting evolutional conservation in function (Loria et al., 2003). While the authors did not determine *unc-75*'s splicing targets, they proposed *unc-75* might act to control synaptic neurotransmission by regulating mRNA splicing in the nervous system.

Several groups have recently taken advantage of new tools to identify the targets of *unc-75* in the nervous system. Through deep sequencing analysis of RNA in both wild type and *unc-75* mutants, Kuroyanagi et al. reported 24 targets of *unc-75*. They also determined a binding motif (G/U)UGUUGUG shared among these targets and confirmed UNC-75 could bind this sequence, which is similar to other studies that identified G/U-rich motifs for the CELF proteins (Dasgupta and Ladd, 2012; Kuroyanagi et al., 2013a). Additionally, these sequences are present upstream of excluded exons, while downstream of included exons of transcripts (Figure 1B) suggesting the location of the UNC-75 binding site could regulate exclusion and inclusion of an exon. In parallel, this group performed a forward genetic screen looking for regulators of isoform specific expression from the gene *unc-32*, which encodes a subunit of vacuolar –type H^+^ ATPases (Pujol et al., 2001; Kuroyanagi et al., 2013b). The *unc-32* gene structure contains two sets of exons alternatively spliced, exon 4 and 7. Exon 4 has 3 possible isoforms—exon 4a, 4b, or 4c—while exon 7 has 2—exon 7a or 7b (Figure 1C). These options for exon 4 and 7 lead to 6 possible transcripts (Figure 1C). mRNAs containing exon 4b and 7a were exclusively expressed in the nervous system, which required *unc-75* (Kuroyanagi et al., 2013b). An *unc-32* mutation that removes the splice site in the exon 4b shows an uncoordinated behavioral phenotype (Pujol et al., 2001). Interestingly, loss of function in *unc-75* suppressed the movement defects of this *unc-32* mutant (Kuroyanagi et al., 2013b). The authors suggest *unc-75(lf)* suppressed the behavioral phenotype of this *unc-32* allele by allowing expression of the other isoforms of *unc-32* in the nervous system to compensate for the loss of exon 4b isoform. Thus, these data provide functional evidence for *unc-75*'s regulation of this specific exon in the nervous system (Kuroyanagi et al., 2013b).

An independent study by Norris et al. has further expanded the list of targets for *unc-75* (Norris et al., 2014). They developed a strategy to generate reporters for alternatively spliced exons in genes previously known to be expressed in the nervous system, such as *mod-1*, a chloride channel, and *dyn-1*, dynamin, and *unc-16*, JIP3 (Norris et al., 2014). A reporter for *unc-16*/JIP3 showed specific expression in the GABAergic motorneurons that depended on the exclusion of exon 16 (Figure 1D). Using this reporter, they performed a forward genetic screen, and identified *unc-75*, as well as another RBP *exc-7* such that in these mutants, the GABAergic specific isoform became ubiquitously expressed in the motor neurons (Figure 1E). They further performed RNA-seq analysis on wild type and *unc-75* mutants and generated gene network predictions for new genes. Among them, they reported that *unc-64*/syntaxin, a protein essential in synaptic vesicle release, is an *unc-75* target (Norris et al., 2014). *unc-64* has two isoforms that differ by the inclusion or exclusion of an exon, and only one of the isoforms can completely rescue *unc-64*'s neuronal phenotypes, suggesting *unc-64*'s splicing must be tightly regulated to ensure proper neuronal function (Norris et al., 2014).

Combinatory interactions of RBPs can allow more complex regulation of mRNA splicing. The *C. elegans* homolog of the ELAV/Hu proteins, *exc-7*, has been found to act in a parallel pathway for mRNA splicing to *unc-75* (Loria et al., 2003; Norris et al., 2014). The ELAV family was first documented in *Drosophila* and named due to the mutant phenotype of embryonic lethal, abnormal visual system (Campos et al., 1985). In humans, the homolog of this family was reported as an antigen in an autoimmune disease and named after the patient whose serum was used, Hu (Graus et al., 1986). Studies characterized Hu as an RBP homologous to *elav* (Szabo et al., 1991), which led to the name of ELAV/Hu family of RBPs. While *unc-75* and *exc-7* have non-overlapping targets based on RNA-seq analysis, they were also found to bind over 40 of the same transcripts, including *unc-16*/JIP3 (Norris et al., 2014). As the *unc-16* isoform excluding exon 16 is expressed specifically in the GABAergic motor neurons, the isoform including exon 16 is expressed in both cholinergic and GABAergic motor neurons (Figure 1D). *unc-75* and *exc-7* are both expressed in the cholinergic neurons, while only *unc-75* is expressed the GABAergic neurons (Loria et al., 2003). Due to these expression patterns in the motor neurons, they were suggested to act together to ensure proper expression of the *unc-16* isoform in cholinergic motor while *unc-75* by itself regulates *unc-16* expression in the GABAergic motor neurons (Norris et al., 2014). Thus, multiple RBPs may act in concert to direct isoform expression from same gene in a cell-specific manner.

AMPA-type glutamate receptors mediate rapid excitatory neurotransmission from worm to human (Bassani et al., 2013). In *C. elegans*, *glr-1* and *glr-2* encode AMPA-type glutamate receptors expressed postsynaptic to motor and inter-neurons, including the command interneurons responsible for regulating the locomotor circuit (Brockie et al., 2001; Mellem et al., 2002; Juo and Kaplan, 2004). To examine the expression pattern and regulation of the AMPA receptor in the AVE interneurons, Wang et al. designed a genomic fusion reporter for *glr-1* and performed a forward genetic screen to isolate mutations that disrupted the localization of receptor (Wang et al., 2010). They identified *grld-1(lf)* mutants as they showed decreased fluorescence intensity of the *glr-1* reporter and a reduction in a response to a nose-touch assay, a behavioral test dependent on the AVE interneuron (Wang et al., 2010). *grld-1* encodes a homolog of the Spen RBP family. Wang et al. found overexpression of a *glr-1* cDNA construct, lacking introns, could rescue the fluorescence intensity of the *glr-1* reporter and partially rescue the nose-touch phenotype (Wang et al., 2010). Additionally, *glr-1* constructs lacking the first two introns partially rescued the fluorescent intensity. Given this data, the authors suggest *grld-1* controls *glr-1* mRNA splicing specifically at introns 1 and 2 (Wang et al., 2010).

The muscleblind-like family (MBNL) consists of RBPs, named after the discovery of the gene in *Drosophila* required in both the muscles and visual system, known to associate with mRNA and regulate splicing (Begemann et al., 1997; Konieczny et al., 2015). In myotonic dystrophy (DM), MBNLs are prevented from binding their targets due to expansions in two genes—dystrophia myotonica protein kinase gene for DM type 1 or CCHC-type zinc finger nucleic acid binding protein gene for DM type 2—that sequester MBNLs in the nucleus (Konieczny et al., 2015). The muscleblind genes are widely expressed in many tissues, yet there has been a focus on their function in the muscles due to the phenotype of myotonic dystrophy. In a screen for regulators of synapse formation in the *C. elegans* DA9 motorneuron, Spilker et al. identified a role for a homolog of the muscleblind family genes, *mbl-1*. The DA9 motorneurons form synapses in the dorsal nerve cord, visualized by a fluorescent-tagged synaptic vesicle marker RAB-3 (Spilker et al., 2012). They found MBL-1 localized to nuclei in the ventral nerve cord. In the *mbl-1* mutant, presynaptic proteins localization was spatially unrestricted in both the dorsal and ventral nerve cords, but the postsynaptic muscle remained normal. Interestingly, a *mbl-1* genomic construct expressed in the DA9 neurons could rescue this synaptic phenotype while muscle expression failed to rescue thus suggesting a neuronal role for this RBP.

A context-dependent regulation of neuronal transcripts was recently reported by Noma et al. (2014). Loss of function mutants of a conserved E3 ubiquitn ligase, *rpm-1*, display an abnormal synaptic phenotype consisting of fewer and disorganized synapses (Schaefer et al., 2000; Zhen et al., 2000). A well-studied downstream target of *rpm-1* is *dlk-1*, a conserved MAP3K (Nakata et al., 2005). In a suppressor screen for *rpm-1(lf)*, *ess-2*, related to the mammalian ES2/DGCR14 protein, was found to regulate *dlk-1* mRNA when splicing junction was altered. ES2/DGRC14 is a component of the splicesome complex (Hegele et al., 2012). *ess-2(lf)* mutants, by themselves, do not show obvious effects on splicing, but reduce the levels of *dlk-1* mRNA expression in a mutant in which the splice site in the third intron of *dlk-1* was mutated. *ess-2(lf)* was also found to act similarly on an allele of *dpy-10*, a cuticle collagen, which changes a splice acceptor site leading to unspliced and spliced transcripts (Aroian et al., 1993). These data suggest *ess-2* functions in splicing in certain contexts, such as when there are non-canonical or mutated splice sites.

The power of forward genetic screening also resides in the discovery of new genes. From a mutant isolated in a screen for genes functioning in GABAergic neuron synapse formation (Zhen and Jin, 1999), Wang et al. reported the identification of *syd-9* (Wang et al., 2006). SYD-9 protein contains C2H2 zinc-finger domains and has a nuclear speckle-like expression in muscles and neurons, similar to other splicing factors. C2H2 zinc-finger proteins are generally known to bind DNA but have also been found to interact with RNA (Razin et al., 2012). In *syd-9* mutant, several presynaptic proteins, including synaptobrevin, were disorganized and animals exhibited abnormal electrophysiological recordings, suggesting impairment in synaptic vesicle release (Wang et al., 2006). Additionally, the authors observed specific genetic interactions of *syd-9* with sets of genes involved in synaptic vesicle (SV) endocytosis, such as synaptojanin and endophilin. Consistent with a model that the abnormal neurotransmission may result from problems in endocytosis, Wang and colleagues found through electron microscopy decreased number of SVs, altered distribution of SVs away from the active zone, and vesicle accumulations in the presynaptic terminal (Wang et al., 2006). The authors suggest that *syd-9* can regulate mRNA processing of these endocytosis genes. The conservation of the C2H2 domains of SYD-9 extends to its homologs in humans (Grondin et al., 1997; Kim et al., 2005). Interestingly, these human proteins, Glis2 and ZNF74, also display similar nuclear speckle-like localization (Grondin et al., 1997; Kim et al., 2005), which suggests functional conservation for this newly discovered gene in higher animals.

## Regulatory nuclear polyadenylation contributes to specific pathways in nervous system development

**Figure 2:**
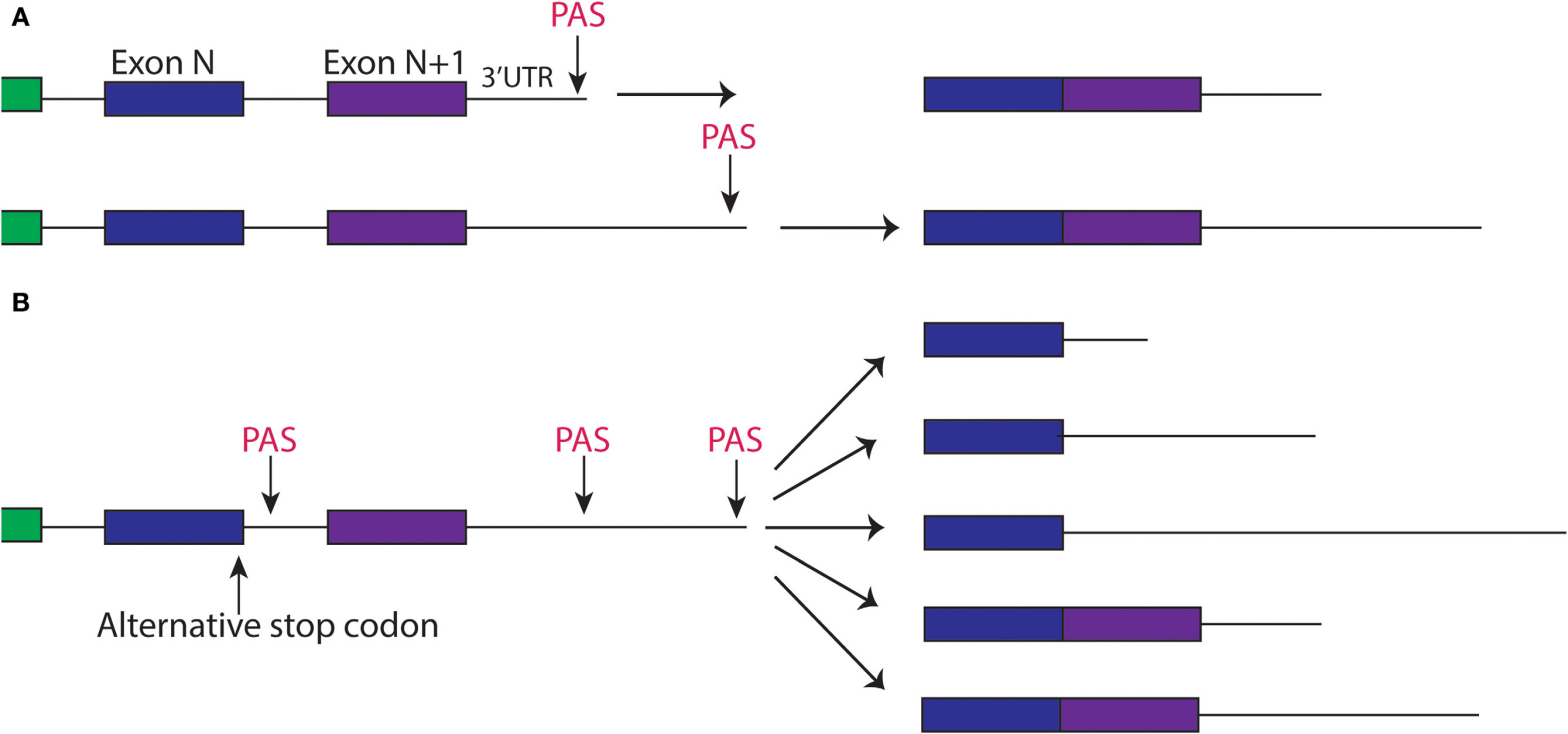
**Alternative splicing and polyadyenylation signals can produce multiple different 3′UTR isoforms of the same gene**. **(A)** Two different PAS sites in the 3′UTR create two different isoforms of transcript, one with a short 3′UTR and one with a longer 3′UTR. **(B)** Alternative splicing of a gene, paired with alternative polyadenylation can create different 3′UTRs that may include the sequence of an exon.

In the nucleus, a key step in parallel to pre-mRNA splicing is 3′-end processing, which leads to formation of 3′-untranslated region and addition of polyadenylates. Production of the 3′-UTR from a pre-mRNA transcript is a multi-step process that includes cleavage at the end, followed by the addition of a polyA tail by the polyA adenylation complex. Nuclear processing of transcripts may result in mRNAs with several 3′UTRs that impart tissue and other types of specificity (Figure 2). In *C. elegans*, the polyadenylation pathway was identified to also affect neuronal development. In a genetic screen for regulators of synapse formation by Van Epps et al. the gene *sydn-1* was found to regulate synapse and axon development such that *sydn-1(lf)* mutants displayed abnormal synapses and ectopic axon branches (Van Epps et al., 2010). SYDN-1 protein appears to be specific among nematodes and is localized to the nucleus. Through a suppressor screen the authors further identified members of the polyadenylation machinery downstream of *sydn-1*, suggesting *sydn-1* inhibits nuclear polyadenylation in the nervous system for proper development (Van Epps et al., 2010). Polyadenylation factors can have temporal and tissue specific regulation of transcripts. In fact, not all of the components of the PolyA complex suppressed *sydn-1(lf)*, which implies specific roles for certain members of this machinery in the nervous system (Van Epps et al., 2010). Thus, it appears that *sydn-1* negatively regulates specific RBPs to ensure proper development of the nervous system.

## Regulation via 3′UTR and small RNA pathways in olfaction

The interaction of RBPs with the 3′UTR of mRNA can serve many functions, such as regulating mRNA's localization, stability and/or translation. microRNAs, bound to argonautes, can directly bind to downstream targets through the targets' 3′UTRs. Studies have discovered that the 3′UTR regulation also extends to the nervous system, including in *C. elegans*. These types of mechanisms can provide tight temporal and spatial control for synaptic plasticity, development and regeneration.

**Figure 3:**
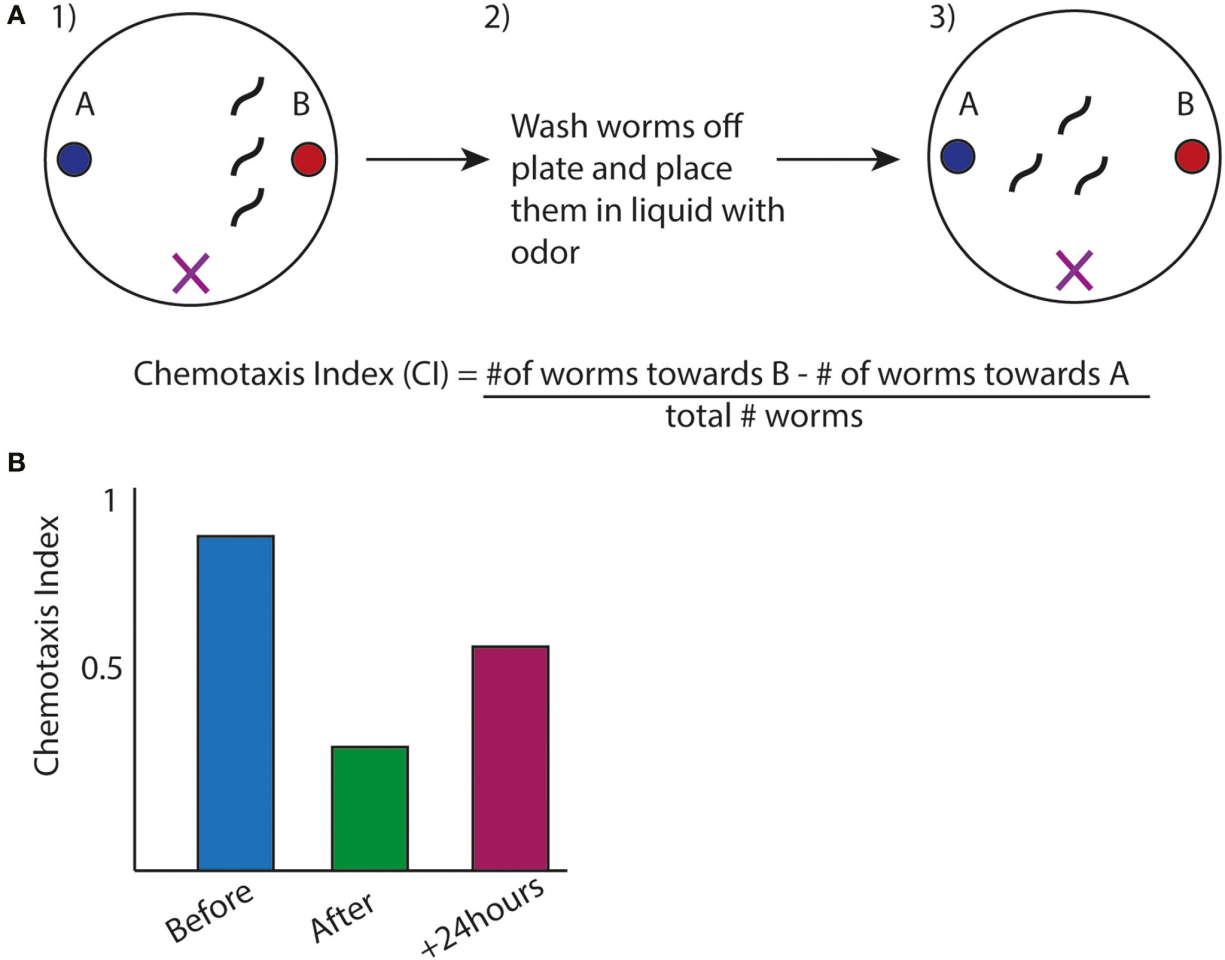
**Olfactory Adaptation Assay used in *C. elegans*. (A)** (1) Animals are placed inbetween two odors (denoted by X), one which they are attracted to (labeled B). (2) Worms are exposed to odor B while starved (3) Animals are placed back onto plates with the same design as in (1). Movement toward the order is measured by a chemotaxis index (CI). The formula is shown (Colbert and Bargmann, 1995). **(B)** Representative graph of chemotaxis index of wild type animals after before, after, and 24 h. after olfactory adaptation assay.

A classical assay in *C. elegans* is olfactory adaptation that is a form of synaptic plasticity allowing the nervous system to strengthen or weaken synapses in response to stimuli, which is thought to underlie memory formation. In this assay animals are tested for their preference for an odor after exposure to the same order (Figure 3A). Wild type animals, after odor exposure, will display avoidance for the trained odor, which fades over time and can be quantified by a chemotaxis index (Figures 3A,B). This behavior depends on the AWC sensory neuron (Bargmann et al., 1993). A series of studies have identified a cGMP-dependent protein kinase, *egl-4* that is expressed in the AWC neuron, to be required in this type of synaptic plasticity and further revealed a regulatory mechanism involving *egl-4*'s 3′UTR (L'Etoile et al., 2002). L'Etoile et al. isolated *egl-4* mutants through a forward genetic screen for suppressors of a dauer phenotype, a specific state the animals enter under starvation or particular stressful conditions, associated with impaired chemotaxis. Interestingly, among alleles of *egl-4*, they found several mutations within *egl-4*'s 3′UTR, suggesting this 3′UTR was important for adaptation. Through 3′UTR sequence analysis of these point mutations, Kaye et al. identified sequences similar to the Nanos Response element within these nucleotides (Kaye et al., 2009). The Nanos Response Element (NRE) is defined based on studies in *Drosophila* embryo where NRE elements are bound by members of the PUF family of RBPs for the proper patterning of the embryo (Wharton and Struhl, 1991). Kaye et al. therefore tested the worm homologs of the PUF family in olfactory adaptation and found that a member of this family, *fbf-1*, was necessary for olfactory adaption to several odors. To address the possibility that *fbf-1* regulated translation of *egl-4* through its 3′UTR, they used a photoconvertible reporter tagged to *egl-4*'s 3′UTR expressed in the AWC neuron. In wild-type animals, new protein synthesis could be visualized after photoconversion in the AWC. This protein translation was absent in *fbf-1(lf)* mutants indicating *fbf-1* promoted translation of the reporter through *egl-4*'s 3′UTR (Kaye et al., 2009). Furthermore, Kaye and colleagues showed that this new protein synthesis correlated with olfactory adaptation: animals with increased protein synthesis of the reporter after the olfactory adaptation assay displayed decreased chemotaxis toward the odor. This pattern of protein synthesis was absent in a reporter with the NRE sequence mutated, suggesting this correlation depended on *fbf*-1's binding site in the 3′UTR. While the PUF family of proteins usually repress translation based on studies in *S. cerevisiae*, *Drosophila* and *C. elegans* (Miller and Olivas, 2011), in this context it appears *fbf-1* promotes translation of *egl-4* for olfactory adaptation.

In addition to the PUF family of RBPs, the small RNA pathways have been identified to have roles in olfactory adaptation. Juang et al. found components of the small endogenous nuclear RNAi pathway that mediate this response (Juang et al., 2013). Loss of function mutations in multiple genes, such as Argonaute, Dicer, and siRNA 3′–5′ exonuclease, fail to adapt to odors (Juang et al., 2013). It was previously shown that the gene *odr-1* is guanyl cyclase specifically expressed in a subset of chemosensory neurons (Bargmann et al., 1993; Yu et al., 1997; L'Etoile and Bargmann, 2000). In this study, they observed that the *odr-1* locus can produce small RNAs known as 22GRNAs and found these 22GRNAs were transcribed from the *odr-1* locus after adaptation. 22GRNAs are small RNAs transcribed from the locus of genes that function in silencing of DNA such as transposons (Gu et al., 2009). This upregulation of *odr-1* derived 22GRNAs depended on the siRNA 3′–5′ exonuclease, *mut-7* (Juang et al., 2013). Given the observation *odr-1* mRNA levels decreased after adaptation, one possibility is the 22GRNAs inhibit *odr-1* transcription. *Hpl-1* and *hpl-2* are the *C. elegans* homologs of the heterochromatin proteins that recognize and bind to specific chromatin marks and repress transcription (Studencka et al., 2012). Juang and colleagues found increased *hpl-2* occupancy at the *odr-1* locus after adaptation that depended on the presence of *mut-7*, providing a mechanism for decreased *odr-1* mRNA after adaptation. Thus, the authors suggest that this small endogenous RNAi pathway can mediate the local regulation of genes for odor adaptation.

Along with the formation of the olfactory memory, a recent study suggests mechanisms involving the 3′UTR of mRNAs can mediate memory loss. Hadziselimovic et al. examined, through a candidate screen, roles for genes interacting with the cytoskeleton for formation and retention of memory (Hadziselimovic et al., 2014). Using the olfactory adaptation assay, they found the RBP Musashi, *msi-1*, was necessary for memory loss after odor adaptation (Hadziselimovic et al., 2014). Musashi proteins consist of two RRMs and are known to be translational repressors (Yoda et al., 2000). Hadziselimovic and colleagues demonstrated that *msi-1* can downregulate the translation of members of the Arp2/3 Complex, a protein structure involved in actin branching, by observing increased fluorescence in reporters for the 3′UTR of certain Arp2/3 components (*arx-1, -2, -3)* in the *msi-1(lf)* (Hadziselimovic et al., 2014). Furthermore, the authors showed a direct interaction between Musashi and the 3′UTRs of the Arp2/3 Complex through immunoprecipitation of MSI-1 and the *arx* mRNAs (Hadziselimovic et al., 2014). They suggest a model in which Musashi regulates part of the actin capping and branching process through the 3′UTR of members of the Arp2/3 Complex to modulate memory retention.

## miRNA in development plasticity

In *C. elegans* larval development, a group of motor neurons, called the DD neurons, undergo synaptic remodeling. They form synapses first on the ventral muscles, which are removed and reformed onto the dorsal muscles after the first-stage larval(L1) (White et al., 1978). This process is important for the nematodes' locomotion as it establishes a circuit of excitation and inhibition of the body wall muscles to allow the movement in a sinusoidal pattern.

A variety of factors, including microRNAs, transcription factors and synaptic activity, influence DD remodeling (Hallam and Jin, 1998; Thompson-Peer et al., 2012). The timing to initiate DD remodeling depends on a heterochronic gene, *lin-14*, as *lin-14(lf)* mutants displayed precocious synaptic remodeling (Hallam and Jin, 1998). *lin-14* was already known as a target of the microRNA *lin-4* (Lee et al., 1993; Wightman et al., 1993), and this modulation of *lin-14* would provide a tight temporal control of its expression. An additional study implicated another target of the microRNA pathway in DD remodeling. Thompson-Peer et al. demonstrated that the C2H2 zinc finger transcription factor *hbl-1*, the *C. elegans* homolog of hunchback, is important for this remodeling process (Thompson-Peer et al., 2012). *Hbl-1* mutants delayed DD remodeling while the microRNA *mir-84*, remodeled precociously compared to wildtype. The *mir-84* developmental phenotype was suppressed by the *hbl-1* mutation, suggesting this microRNA pathway controls synaptic remodeling by negatively regulating *hbl-1* (Thompson-Peer et al., 2012). By examining a reporter for *hbl-1*'s 3′UTR, Thompson-Peer and colleagues observed increased fluorescence in the *mir-84* mutant, hinting *mir-84* down-regulated *hbl-1* through its 3′UTR (Thompson-Peer et al., 2012). Thus, this study identified *hbl-1* as a microRNA target for synaptic remodeling.

## miRNA in dauer formation

MicroRNAs can affect other developmental processes in the nervous system, such as dauer formation. For example, Than et al. found roles for neuronal microRNAs in dauer formation (Than et al., 2013). The authors found that neuronally enriched microRNAs, *mir-81/82*, *mir-234*, and *mir-124* loss of function mutants enhanced dauer development with *unc-3(lf)* (Than et al., 2013). UNC-3 is a *C. elegans* homolog of the Collier/Olf-1/Early B-cell Factor (COE) transcription factor family shown to inhibit dauer formation (Prasad et al., 1998). This dauer formation was enhanced when *unc-3(lf)* was paired with the GW182 homolog, *ain-1(lf)*, suggesting microRNA involvement. However, the individual mutants of the microRNAs and *unc*-3*(lf)* could not completely recapitulate the percentage of dauer formation observed in *unc-3; ain-1*, suggesting that multiple microRNAs contribute to the dauer phenotype (Than et al., 2013). With further improvement, such efforts will aid the studies of cell-specific expression of microRNAs acting by themselves and combinatorially with other microRNAs.

## miRNA in synaptic transmission

The microRNA pathway has also been implicated in synaptic transmission. In *C. elegans*, the conserved *miR-1* family of microRNAs has been useful to study, as mutations are viable, whereas in other organisms mutations are lethal. While *C. elegans mir-1* is expressed in the muscles and not in the nervous system, Simon et al. found *mir-1* regulated a signal passed from the muscle to neuron to modulate synaptic transmission (Simon et al., 2008). The authors identified MEF-2, a transcription factor, as a target of *mir-1* in the muscle and suggest a model where MEF-2's transcriptional targets affect the presynaptic terminals of neurons at the neuromuscular junction (NMJ) by inhibiting neurotransmitter release (Simon et al., 2008). In another paper looking at possible targets of *mir-1* in synaptic transmission, two synaptic adhesion proteins, neurexin and neuroligin were examined. Both genes were found to suppress *mir-1*'s phenotype of decreased acetylcholine release, suggesting these two adhesion proteins transmit the retrograde signal to decrease neurotransmission (Hu et al., 2012).

## Roles of miRNA and mRNA regulation in axon regeneration

In the adult nervous system, the ability to quickly respond to stimuli such as an injured neuron is quite essential. 3′UTRs are known to mediate mRNA localization and translation in dendrites and axons and may provide a rapid response to axon injury (Andreassi and Riccio, 2009). Recent studies have shown axon regeneration after injury can depend on mRNA 3′UTRs and RBPs.

MicroRNAs are important mediators of mRNA regulation in several species for axon regeneration (Yu et al., 1997; Williams et al., 2009; Liu et al., 2013; Zou et al., 2013). In the nervous system, there is an age-dependent decline in regeneration across all species studied and which several groups have tried to address. Zou et al. demonstrated in the *C. elegans* AVM neuron that this decline could be mediated by microRNA processing pathway and its targets (Zou et al., 2013). Animals mutant for the argonaute gene *alg-1* showed enhanced regeneration in older animals compared to wild type controls. Since Argonautes are guided to their targets by microRNAs, the authors used RT-PCR over the larval and adult stages to determine which microRNAs could mediate this phenotype. The microRNA *let-7* increased in expression as the animal aged and its loss of function mutant also showed enhanced regeneration (Zou et al., 2013). The study followed up by examining known targets of *let-7* and found a member of the TRIM family proteins, *lin-41*, as a downstream target in axon regeneration. Using a reporter tagged to *lin-41*'s 3′UTR, the group showed decreased intensity of the reporter in aged animals, which correlated with *let-7*'s increased expression (Zou et al., 2013). In addition, immuoprecipitation showed a direct interaction between LIN-41 and ALG-1 (Zou et al., 2013). Thus, increased *let-7* expression in aged animals could decrease nerve regeneration by binding and down-regulating LIN-41 through *lin-41*'s 3′UTR. Interestingly, in a different touch neuron, the PLM, *alg-1(lf)* inhibits adult axon regeneration (Chen et al., 2011), suggesting cell-context and stage-dependent regulation by miRNAs.

In PLM mechanosensory neurons, Yan et al. reported the 3′UTR of a transcription factor important for axon regeneration (Yan et al., 2009). This transcription factor, *cebp-1*, is the homolog of the CCAAT/Enhancer Binding Protein family (C/EBP) and found to be necessary for axon regeneration (Yan et al., 2009). *cebp-1* was identified through a suppressor screen for downstream targets of the conserved ubiquitn E3 ligase, *rpm-1*, which also was found to negatively regulate *cebp-1* mRNA levels (Yan et al., 2009). Two key pieces of data implicated the 3′UTR of *cebp-1* as important for its regulation. First, transgenes expressing *cebp-1* without its 3′UTR partially mimicked *rpm-1(lf)*'s phenotype, suggesting *rpm-1* negatively regulated *cebp-1* through its 3′UTR. Second, using a photoconvertible protein tagged to *cebp-1*'s 3′UTR, they showed upregulation of protein synthesis in the distal axon 2 h after axotomy. When examining a similar reporter tagged to a control 3′UTR this increase in fluorescence in the distal axon was absent. Taken together, these data suggest upregulation of *cebp-1* through its 3′UTR after injury is important for the regenerative response, illustrating a function for the 3′UTR in the adult nervous system. Though so far there are few studies of RBPs and microRNAs in *C. elegans* in axon regeneration, they all implicate roles for RBPs and microRNAs in controlling local responses to injury and regeneration.

## Conclusions and perspectives

The complexity of the nervous system poses many questions about how it develops, grows and maintains its connections. Studying RNA regulation will identify key mechanisms that can address these questions. *C. elegans* has helped advance the field by providing genetic tools to dissect RNA pathways, specifically in the nervous system. Since many RBPs are conserved, their function and mechanism of regulation in *C. elegans* may likely be the same in humans. Additionally, *in vivo* studies in *C. elegans* have displayed the importance of RBPs in mRNA splicing, mRNA 3′-end processing and microRNA pathway, and their resulting effects on synaptic plasticity, synapse formation and axon regeneration. These studies have allowed single-neuron and global dissection of these interactions to highlight the complexity of the nervous system. Future studies of mRNA in the nervous system of *C. elegans* will continue to build a deeper understanding of the complex regulations by RBPs.

### Conflict of interest statement

The authors declare that the research was conducted in the absence of any commercial or financial relationships that could be construed as a potential conflict of interest.
